# Stromal vascular fraction in the treatment of myositis

**DOI:** 10.1038/s41420-023-01605-9

**Published:** 2023-09-19

**Authors:** S. Gandolfi, B. Pileyre, L. Drouot, I. Dubus, I. Auquit-Auckbur, J. Martinet

**Affiliations:** 1 Univ Rouen Normandie, INSERM U1234, FOCIS Center of Excellence PAn’THER, F-76000 Rouen, France; 2 Toulouse University Hospital, Department of Plastic and Reconstructive Surgery, F-31000 Toulouse, France; 3 Centre Henri Becquerel, Department of Pharmacy, F-76000 Rouen, France; 4 Univ Rouen Normandie, INSERM U1234, FOCIS Center of Excellence PAn’THER, CHU Rouen, Department of Plastic, Reconstructive and Hand Surgery, F-76000 Rouen, France; 5 Univ Rouen Normandie, INSERM U1234, FOCIS Center of Excellence PAn’THER, CHU Rouen, Department of Immunology and Biotherapy, F-76000 Rouen, France

**Keywords:** Mesenchymal stem cells, Autoimmune diseases, Autoimmunity, Drug development

## Abstract

Muscle regeneration is a physiological process that converts satellite cells into mature myotubes under the influence of an inflammatory environment progressively replaced by an anti-inflammatory environment, with precise crosstalk between immune and muscular cells. If the succession of these phases is disturbed, the immune system can sometimes become auto-reactive, leading to chronic muscular inflammatory diseases, such as myositis. The triggers of these autoimmune myopathies remain mostly unknown, but the main mechanisms of pathogenesis are partially understood. They involve chronic inflammation, which could be associated with an auto-reactive immune response, and gradually with a decrease in the regenerative capacities of the muscle, leading to its degeneration, fibrosis and vascular architecture deterioration. Immunosuppressive treatments can block the first part of the process, but sometimes muscle remains weakened, or even still deteriorates, due to the exhaustion of its capacities. For patients refractory to immunosuppressive therapies, mesenchymal stem cells have shown interesting effects but their use is limited by their availability. Stromal vascular fraction, which can easily be extracted from adipose tissue, has shown good tolerance and possible therapeutic benefits in several degenerative and autoimmune diseases. However, despite the increasing use of stromal vascular fraction, the therapeutically active components within this heterogeneous cellular product are ill-defined and the mechanisms by which this therapy might be active remain insufficiently understood. We review herein the current knowledge on the mechanisms of action of stromal vascular fraction and hypothesise on how it could potentially respond to some of the unmet treatment needs of refractory myositis.

## Facts

- Muscle regeneration involves a sequence of tissue repair mechanisms regulated by both pro- and anti-inflammatory immune cells.
- Myositis pathomechanisms are not fully understood but may result from chronic exposure to immune cells and cytokines, leading to destruction and mis-repair of muscle, with fibrosis and vascular architecture disturbance.
- Therapeutic options for inflammatory myopathies are predominantly based on immunosuppressive treatments, which are sometimes insufficient to regulate the different features of these complex pathologies.
- Adipose tissue-derived stem cells and stromal vascular fraction have immunomodulatory, anti-fibrotic, proangiogenic and regenerative properties that could be exploited for the treatment of refractory or relapsed myositis.
- However, the mechanisms behind these effects are still insufficiently understood and more preclinical studies are required before their clinical use.

## Open questions

- Does repeated acute muscular destruction trigger chronical autoimmune inflammation, or is auto-immunity responsible for muscle destruction in myositis?
- In myositis, how does chronicle inflammation affect adipose-derived cells capacities?
- Will adipose stem cells replace muscle stem cells and directly participate in tissue regeneration, or rather have a supporting role for immune and local stem cells?

## Introduction

The mechanisms of muscle regeneration following injury are now well-known and involve both muscle and immune cells, through a regulated process [1]. A disruption of this process may be the cause of chronic inflammation and a failure of muscle regeneration, which can lead to autoimmune diseases such as myositis. Myositis, also called idiopathic inflammatory myopathies, represents a group of immune-mediated diseases, including Polymyositis (PM), Dermatomyositis (DM), Immune-Mediated Necrotising Myositis (IMNM), Inclusion Body Myositis (IBM) and overlap myositis [2]. They are clinically characterised by muscle weakness, and histologically by the presence of varying levels of myofibre necrosis and leucocyte infiltrates in muscles [3]. Predominantly muscular, some forms of myositis can also be associated with other manifestations, such as Interstitial Lung Disease (ILD), skin ulcers or Raynaud’s phenomenon. Corticosteroids and immunosuppressive drugs are commonly used but may be ineffective in some patients or even aggravating due to their possible side effects [4–6].

For these patients, cell therapies have sometimes shown long-term beneficial effects. Autologous hematopoietic stem cell transplants, which have been used to replace auto-reactive immune systems, have improved patients’ conditions, and even allowed some of them to enter into remission. These therapies generally seem to be safe, but can be complicated by severe or even life-threatening iatrogenic infections due to myeloablative conditioning regimens [7–10]. More recently, the discovery of the immunomodulatory effects of Mesenchymal Stem Cells (MSC) from Bone Marrow (BM-MSC) or Umbilical Cord Blood (UC-MSC), in addition to their well-known regenerative effect, has led to their use for the treatment of patients with refractory autoimmune diseases, including myositis [11]. If these types of stem cells seem safer, the invasiveness of the harvesting and the low rate of stem cells recovered from these sources remain important limitations to their use.

Adipose tissue is another source of MSC. They can be extracted safely and in large quantities from a lipoaspirate by enzymatic digestion or mechanical isolation for Stromal Vascular Fraction (SVF), followed by replicative cultures of adherent cells for Adipose-Derived Stem Cells (ADSC) [12]. If ADSC are known to possess immunomodulatory, proangiogenic, anti-fibrotic and regenerative capacities like MSC from other tissues [13], SVF share similar properties and is easier to prepare [14]. However, the therapeutically active components within this heterogeneous cellular product are not well defined, and the mechanisms responsible for its activity remain insufficiently understood.

In order to evaluate the potential of SVF as a treatment for refractory myositis, we first summarise here the physiological mechanisms of muscle regeneration and the pathological mechanisms involved in myositis. Next, we address the main features of these diseases through the known mechanisms of action of this cell therapy. Last, we discuss its clinical relevance by analysing results from various clinical trials.

### Physiological muscle regeneration

Muscle regeneration after trauma is a process that involves both immune and muscular cells in order to restore normal muscle function. At first, satellite cell activation and proliferation accompanied by inflammation, followed by a progressive decrease of inflammation under the influence of anti-inflammatory cells, which stimulate muscle progenitor cell differentiation and tissue remodelling [15].

At the earliest stage of regeneration after injury, muscle damaged cells release Damage-Associated Molecular Patterns (DAMP) [16], which lead through Toll Like Receptor (TLR) to the activation and infiltration of immune cells, mostly mast cells [17] and neutrophils [18]. These cells start to clear the damaged myofibres and secrete pro-inflammatory cytokines (mostly IL-1, IL-6, IL-8 and TNFα). The pro-inflammatory signal spreads and after 24 h, macrophages can be observed at the lesion site [19]. They are mostly involved in the elimination of damaged muscular cells by the production of Reactive Oxygen Species (ROS), through the increased expression of Inducible Nitric Oxide Synthase (iNOS), and phagocytosis. Like neutrophils, they secrete a large amount of cytokines (mostly TNFα, IL-6, and IL-1β) which triggers a positive feedback loop between neutrophil and macrophage recruitment and production of cytotoxic substances, but also T-cell recruitment [20].

Around three days after injury, both CD8^+^ and CD4^+^ T cells appear at the lesion site and can be detected for up to ten days [21]. CD8^+^ T cells pursue the task of macrophages and neutrophils, by releasing many cytokines which amplify the recruitment of leucocytes and by acting on extracellular matrix remodelling to speed up cellular debris elimination [22]. CD4^+^ T cells seem to be recruited a little later than CD8^+^ T cells [23]. They differentiate preferentially into Th1 cells, which maintain macrophage recruitment and pro-inflammatory polarisation through the production of cytokines (IL-1β, TNFα and IFNγ).

The large amount of secreted cytokines modulates the environment of the injured site and triggers muscular regeneration [1]. Fu et al. demonstrated that the cytokines secreted by T cells promote satellite cell proliferation in vitro and in vivo [24]. Indeed, TNFα is known to attract satellite cells to the damaged site and to enhance their proliferation through the activation of NF-κB signalling and the p38 pathway [25, 26]. The importance of TNFα secretion in response to muscular damage has been demonstrated in vivo in TNFα or TNF-receptor knockout mice, which show severe muscular regeneration defects [27, 28]. TNFα is associated with other cytokines, such as IL-6, secreted by both immune and muscle cells, and IL-1β, which maintains the proliferation but also stimulates the differentiation of satellite cells into myoblasts [29, 30]. The importance of IL-1β has also been demonstrated in IL-1β knockout mice, which present a slowdown of satellite cell differentiation, shown by a reduced expression of myoblast markers MyoD and Myogenin.

When the clearing of damaged cells ends, the naive T cells recruited polarise into Th2 cells, which release anti-inflammatory cytokines (IL-4 and IL-13) [31]. These cytokines stimulate myoblast fusion and Fibro–Adipogenic Progenitor (FAP) proliferation [32]. After activation and differentiation, FAP are another source of myogenic factor like Wnt family members, IL-6 and Insulin-like Grow-Factor 1 (IGF1), which enhance satellite cell proliferation and myoblast differentiation and fusion [33–35]. They also release IL-33, which participates in the activation of muscle Treg cells and the recruitment of circulatory Treg cells, which are genetically and functionally distinct [36]. Indeed, in addition to regulating and reducing the inflammatory environment, muscle Treg cells play an important role in regeneration through amphiregulin secretion [37, 38]. The change of inflammatory environment is mainly based on the change of macrophage populations from pro-inflammatory to anti-inflammatory, under the influence of Th2 and Treg cell cytokines [39]. These macrophages produce anti-inflammatory cytokines (IL-4, IL-10 and IL-13), which reduce the local inflammation induced by the lesion and stimulate the differentiation and fusion of myoblasts into myotubes, promoting the late stage of myogenesis [35, 40]. They also release TGFβ, which regulates myotube fusion and prevents the TNFα-induced apoptosis of FAP, inducing their differentiation into matrix-producing cells [41]. These cells also produce growth factors such as IGF1, Hepatocyte Growth Factor (HGF), basic Fibroblast Growth Factor (bFGF), and Vascular Endothelial Growth Factor (VEGF), which stimulate endothelial cells and contribute to bringing together endothelial and satellite cells [42]. The proximity and crosstalk between muscular, mesenchymal and endothelial cells are essential for both myoangiogenesis and total muscle recovery [43].

### Defective muscle regeneration during myositis

**Fig. 1:**
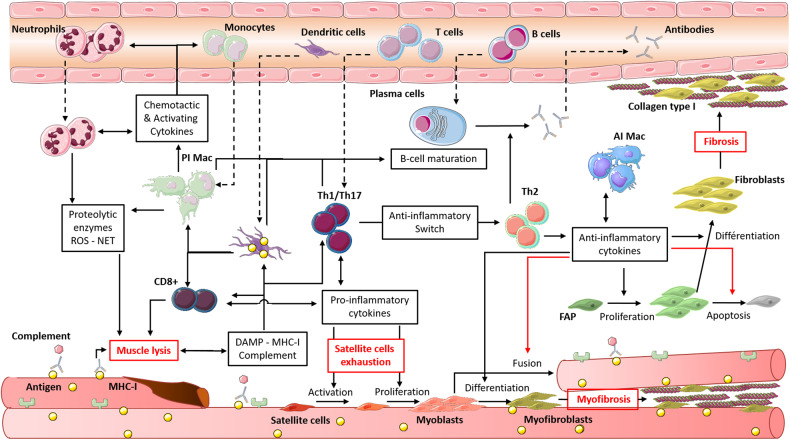
Skeletal muscle degeneration in myositis is dependent on both pro- and anti-inflammatory cells. Depending on the muscle area, adaptive immune cells maintain a pro- or anti-inflammatory environment through cytokine secretion. The pro-inflammatory environment leads to an accumulation of myeloid cells, mostly neutrophils and macrophages, which secrete proteolytic enzymes and ROS by iNOS overexpression. These molecules degrade myotubes which release signals that stoke pro-inflammatory cells (DAMP). Moreover, myofibres over express muscular antigen and MHC class I, which stimulate even more adaptive cells, directly or via specific antibodies and/or complement system. Plasma cells are activated by a B-cell maturation environment generated by BAFF expression by DC and T cells and secrete specific auto-antibodies. Pro-inflammatory cytokines also stimulate proliferation and activation of satellite cells, until exhaustion. The anti-inflammatory environment replaces pro-inflammatory macrophages (PI Mac) with anti-inflammatory macrophages (AI Mac), which secrete large amounts of TGFβ and other anti-inflammatory cytokines. These cytokines induce myofibrosis through myoblast differentiation into myofibroblast, by inhibiting myotube fusion, and fibrosis through fibroblast differentiation from FAP, by inhibiting TNFα-induced apoptosis of these cells. Fibrosis accumulation leads to muscular and vascular disturbance, and to a loss of strength.

The myoregeneration process relies on the presence of functional satellite cells in the muscle and the environmental agents that control at different phases the proliferation, differentiation and fusion of these cells into myotubes [44]. Among these stimuli, secretion by immune cells of pro- then anti-inflammatory cytokines plays an important role. In the case of myositis, a chronic activation of innate and adaptive immune cells which can recognise auto-antigens is observed and these cells, normally transient, remain in the muscle (Fig. 1). Neutrophil persistence in muscle infiltrates has been demonstrated in myositis muscle, as well as neutrophil participation in the destruction of muscle fibres through the release of proteolytic enzymes and the formation of neutrophil extracellular traps [45, 46]. Further, this extracellular formation seems to be induced by the presence of myositis-associated antibodies. Reimann et al. showed an increase in pro-inflammatory macrophage density in muscle, through iNOS expression correlated with a high expression of macrophage migration inhibitory factor, a cytokine with anti-apoptotic, proliferative and chemotactic effects secreted by macrophages, T cells and muscle fibres [47]. Myositis patients present a strong Th1 response, with an increased expression of IFNγ, IL-1β and TNFα [48, 49]. This Th1 response leads to the induction and maintenance of pro-inflammatory macrophages and thus amplifies muscle destruction [50]. A type 1 IFN signature was observed in the blood of patients with DM or PM and correlated with disease activity [51]. A recent study showed that the activation of type 1 IFN pathway in muscle cells in vitro induced myotube atrophy and impaired endothelial cell angiogenesis, features that were observed in DM [52]. Muscle destruction is also due to the abnormal expression of Major Histocompatibility Complex type I (MHC-I) on the myocyte surface, induced by IFNγ and IL-1β [53]. This expression is also induced by IL-17, produced by Th17 cells, which potentiates the effects of IL-1β. Some studies have shown that overexpression of MHC-I in muscle can induce myopathy through both immunological, with CD8^+^-mediated cytotoxicity, and nonimmunological mechanisms [54, 55]. Thereby, both CD4^+^ and CD8^+^ T cells can be activated by muscle cells, which act directly as antigen presenting cells, but also indirectly through dendritic cells (DC) detected in muscular infiltrate during myositis [56], or TLR stimulation by DAMP [16, 57]. The activation and differentiation of T cells by these cells lead to the formation of a permissive environment for B-cell maturation, evidenced by the presence of CD19^+^ or CD20^+^ B cells and CD138^+^ plasma cells and by the expression of B-cell activating factor [58]. Myositis specific antibodies were identified in more than half of patients [2], and a recent study associated Th1 and Th17 cytokine expression with B-cell aggregation and maturation, through the formation of ectopic lymphoid structures in myositis muscle [59]. The pathogenicity of these antibodies has not always been demonstrated, however they are often correlated with the severity of the myositis and the underlying diseases, like ILD, Raynaud syndrome or cancer [60]. Furthermore for some of them, the mechanism of action has been identified, notably in IMNM, wherein anti-3-Hydroxy-3-methylglutaryl-CoA reductase (HMGCR) and anti-signal recognition protein (SRP) induce myofibre atrophy by increasing IL-6, TNFα and ROS secretion. They also reduce myotube formation by decreasing IL-4 and IL-13 production [61]. This pathogenic effect seems also to involve complement system [62].

Anti-inflammatory cells are also present in myositis muscle and some of them take part in the pathomechanism. If the Th2 cytokine IL-4 overexpressed in myositis seems inversely correlated with the severity of muscular destruction, as evidenced by muscular enzyme levels in the sera [63], anti-inflammatory macrophage cytokines (TGFβ and IL-10) are also highly expressed in myositis and seem to be involved in the pathogenesis [47, 64]. Prolonged exposure to TGFβ and IL-10 is correlated with the duration of myositis via their association with other cytokines, especially in IBM [50, 65]. Prolonged TGFβ1 exposure, for example, is strongly involved in the dysregulation of muscle regeneration in different myopathies, by inhibiting satellite cell activation and differentiation and myocyte fusion, but also by increasing myofibroblast accumulation and fibrosis [66–68]. Muscle fibrosis is also generated by the accumulation of FAP, which escape TNF-induced apoptosis and differentiate into collagen type-1 producing cells (fibroblasts) under TGFβ1 high expression by anti-inflammatory macrophages [41, 69]. This escape from apoptosis could also be due to the expression of immune checkpoint factors (PD-L1, PD-L2 and CD47) by FAP, as shown in a murine model of myositis [70]. Fibrosis can lead, like in IBM, to tissue and capillary architecture disruption and to an increased distance between muscle fibres and capillaries, responsible for hypoxia and oxidative stress [71]. Treg cell functions are also altered in myositis, which leads to the dysregulation of immune response and to the impairment of muscle regeneration. Indeed, Treg dysfunctions have been evidenced in both DM and PM and in several myositis models, which present a more severe disease when Treg cells are depleted. Conversely, the injection of functional Treg cells at the time of immunisation blocked disease progression [72]. On the other hand, Treg cells seem to be fully functional in IBM despite a decreased frequency observed in circulation and muscle [49].

If the pathogenic triggers of myositis have not yet been identified, partially due to a lack of spontaneous animal models, the hypothesis of an immune system defect which is solely responsible or even at the origin of the disease is controversial, especially in IBM [73, 74]. Muscle cells participate in the pathogenesis and aggravation of the disease, through the activation of several deleterious and not fully understood mechanisms, involving endoplasmic reticulum and/or mitochondrial defects. If MHC-I expression on muscle cell surface is often linked to leucocyte infiltration, it has been shown that it can be induced by endoplasmic reticulum stress, and that its presence can be detected before infiltrate and can affect muscle fibre contractibility [75]. Beyond muscle destruction, which stays chronically blocked in pro- and/or anti-inflammatory state without resolution, the impairment of satellite cell capacities to activate and proliferate is also involved in the disease. Myoblasts from IBM patients also present reduced proliferation rate and clonogenicity capacity in vitro when compared to myoblasts from healthy donors, probably due to replicative exhaustion and senescence [76]. This observation was confirmed in vivo, with a lower expression of MyoD [71]. Moreover, the persistence of myogenin expression observed in these patients might reflect an impairment of both myoblast differentiation and muscle fibre maturation.

Myositis represents a heterogeneous group of diseases which involve immune and non-immune mechanisms. In these diseases, the immune system acts on both muscle degeneration and regeneration, with muscle progenitor cell stimulation by cytokines. While suppressing myositis may appear as an effective solution to stop or to slow down the disease, this can be insufficient or even aggravating sometimes, due to non-immune mechanisms or regenerative defects.

### Current therapeutics for myositis patients

Most of the treatments used in myositis target the inflammatory actors. Glucocorticoids are the most current therapeutic approach, alone as a first-line treatment or associated with other immunosuppressive molecules in refractory patients [77]. These associations are more efficient to improve muscle function, and act on myositis-associated diseases like ILD. Moreover, they allow to reduce the corticosteroid effective dose, leading to fewer side effects, especially on muscle [78]. Nevertheless, the use of immunosuppressive molecules is also limited by their own adverse effects, including toxicities on various organs (liver, lung, kidney, heart), risk of infection and metabolic disorders (diabetes, dyslipidemia) [79]. These various side effects are even more problematic in elderly subjects who suffer from numerous co-morbidities. Biological agents have also been tested in myositis: at first, Intravenous (IV) immunoglobulins, considered to present immunomodulatory properties, and then monoclonal antibodies, such as Rituximab (anti-CD20), Tocilizumab (anti-IL-6), Infliximab (anti-TNFα), Alemtuzumab (anti-CD52), Basiliximab (anti-IL-2R) or Sifalimumab (anti-IFNα), or even receptor antagonist Anakinra (anti-IL-1R), JAK inhibitor Tofacitinib and fusion proteins Abatacept or Etanercept, which block CTLA-4 or TNFα pathways, respectively [6, 80, 81]. If these treatments are efficient in most of first- and second-lines refractory to DM and PM, some patients stay refractory or relapse to all of them. Complement inhibition has also been tested in IMNM, with a preventive effect in animals but no significant improvement in management in humans [82, 83]. Further, these treatments seem to bring no benefit and even to exacerbate the disease in IBM patients, who remain in a therapeutic deadlock [4]. In this myositis subtype, new molecules are under evaluation, like Arimoclomol, a Heat Shock Factor 1 activation amplificator, which increases chaperone protein activity and thus misfolding protein repair pathway, or follistatin, a myostatin antagonist, with an adeno-associated virus coding for it injected Intramuscularly (IM) [84]. New monoclonal antibodies targeting myostatin or activin A, which both negatively regulate myogenesis, have been developed but clinical trials in IBM have been recently withdrawn or cancelled for these treatments [85].

Cell therapies are occasionally used to treat patients with refractory PM or DM, but very few cases have been reported. As stated above, hematopoietic stem cell autologous transplantation, used to replace auto-reactive immune system after its depletion, has shown benefits but with a high risk of side effects [7–10]. MSC transplantations, for which no myeloablative treatment is required, have also been tested (Table 1).

**Table 1:** Summarises of clinical reports of myositis treat by MSC.

Study	Treatment	Follow-up	Efficacy	safety
Wang et al. (2011) [85]	IV Allogeneic BM or UC-MSC (*n* = 10) (uncontroled)	1, 2, 3, 6, 12, 18 and 24 months Clinic: Severity (VAS score), manuel muscle test (MMT score) and adverse events (AEs) Biologic: CK, CK-MB	MMT and VAS improved Inflammation reduced on MRI (*n* = 2) Associated symptom improved (ILD, skin ulcers) CK and CK-MB decreased significantly Baseline treatments reduced Relapses (*n* = 3) at 6 or 8 months, second MSC transplant (*n* = 2), with second relapse (*n* = 1) at 3 months	AEs (*n* = 0) Death (*n* = 2) starting by respiratory tract infection, disease deterioration and heart failure or severe myocarditis
Ra et al. (2011) [87]	IV autologous ADSC (*n* = 1) (uncontroled)	3 months Clinic: MMT, quality of life (SF-36) Biologic: in blood (not detailed)	Muscle strength and quality of life improved, allowing patient to step up stairs, to walk holding handrail, to drive by herself No significant change was detected in blood Baseline treatment reduced	Not specified
Lai et al. (2015) [86]	Conventional therapy (*n* = 44) IV Allogeneic UC-MSC (*n* = 37)	1, 3 and 6 months Clinic: Muscle force grade, lung imaging Biologic: CK	Muscle force grade increased in both groups, but higher in UC-MSC group Lung imaging improved CK reduced in both groups, but lower in UC-MSC group	Not specified
Liang et al. (2018) [11]	IV Allogeneic BM or UC-MSC (*n* = 30) (uncontroled)	From 2007 to 2016 Clinic: AEs	Not specified	Hyperacute AEs (*n* = 6): fever, headache, palpitation, facial redness, insomnia, stomach discomfort. Death (*n* = 11): underlying disease (ILD)

The first clinical study using MSC was published in 2011 by Wang et al. and reported the case of 10 patients with refractory PM or DM, according to Bohan and Peter criteria [86]. After treatment by IV allogeneic BM- or UC-MSC, they observed an improvement in muscle strength and clinical score and a reduction of inflammation and muscle degeneration [87]. Transplanted cells were also effective on associated symptoms, notably ILD and skin ulcer, and allowed the reduction of immunosuppressive baseline treatments for all patients. However, three patients relapsed and two patients died from aggravation of a respiratory tract infection. This cohort was followed-up and has grown overtime to thirty patients and safety data were published in 2018. They showed a low frequency of hyperacute adverse events, but reported the death of 11 patients from their underlying diseases, mostly ILD [11].

Lai et al. published the only controlled clinical trial, comparing conventional therapy (6 months of corticosteroids and immunosuppressors) with (*n* = 37 patients) or without (*n* = 44 patients) allogeneic UC-MSC injections in patients with PM or DM [88]. After a follow-up of 6–12 months, the authors demonstrated an improved efficacy of conventional therapy by MSC infusion on both clinical features and muscle strength (MMT score), confirmed by a decrease in plasma creatine kinase level. They also showed an effect on associated ILD, with a reduction of interstitial pulmonary lesions on high resolution CT scan. Clinical and biological controls at 1, 3 and 6 months after transplantation did not reveal any complications.

Only one study reported the case of a patient with refractory and disabling PM treated by four ADSC infusions, leading to an improvement in muscle strength (MMT score) and mobility 3 months after treatment, but no significant change in blood laboratory values [89]. The patient continued corticosteroid treatment at a lower dose.

Pharmaceutical and biological treatments have evolved and now allow to treat most of patients with myositis. After multiple lines and associations of treatment, some patients with PM or DM remain refractory and more often those with IBM. In the latter case, new treatments under evaluation target more specifically muscle degeneration, through metabolic or regulatory pathways, but the expected effect is not always obtained. Conversely, MSC therapies have shown interesting results in myositis, but their harvest and preparation are not simple and clinical studies remain rare. SVF, which has similar effects, could be an alternative, provided its clinical efficacy is proved through robust clinical trials. Two of them are about to start to study the safety of SVF in IBM (NCT04975841, NCT05032131).

### Stromal vascular fraction in clinical practice

**Fig. 2:**
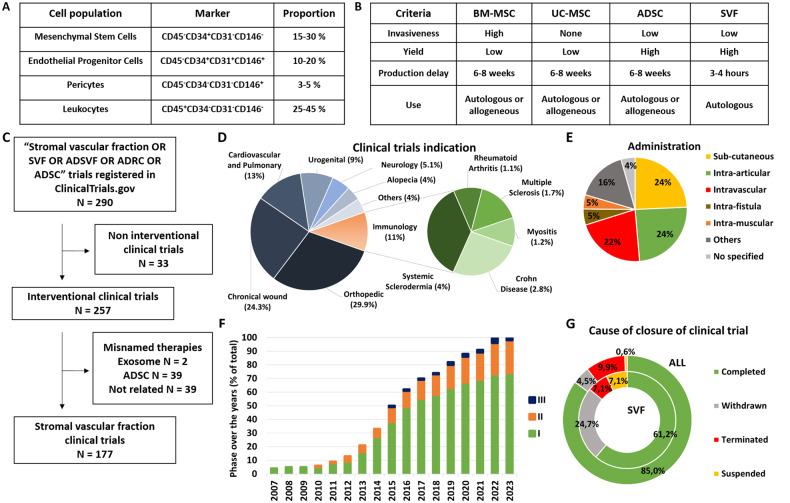
Stromal vascular fraction description and clinical trials. **A** Description of stromal vascular fraction cell population and main molecular marker. **B** Advantages and disadvantages of stromal vascular fraction compared to ADSC or others MSC. **C** Flow diagram research and selection of ClinicalTrial using SVF. Representation of the distribution of clinical trials **D** by indication and sub-indication for immunology, **E** by way or localisation of administration, **F** by phase over the years and **G** by cause of closure.

SVF isolation consists in adipocyte elimination through enzymatic or mechanical procedures [90, 91]. Due to the variety of isolation methods, the International Federation for Adipose Therapeutics and Science (IFATS) and the International Society for Cellular Therapy (ISCT) have released a joint statement that defines phenotypic and functional criteria for the identification of adipose-derived cells, and proposes a general composition for SVF [12]: MSC (15–30%), Endothelial Progenitor Cells (EPC) (10–20%), Pericytes (3–5%) and leucocytes (25–45%) (Fig. 2A). According to the SVF isolation method used, two types of SVF can be defined: cellular SVF, which can be obtained by enzymatic procedure, and tissular SVF, which is mechanically isolated and preserves cell-cell contact and extracellular matrix [92, 93]. Recently, another intermediate type has been described: adipose-derived microvascular fraction which consists in lipoaspirate cells enzymatically digested for a shorter time than cellular SVF, and which contains intact arteriolar, capillary and venous vessel segments [94].

Today, SVF is widely used and frequently compared to ADSC in both regenerative medicine and immune/inflammatory disorders and presents significant advantages. Indeed, SVF extraction is simpler and quicker than ADSC expansion and does not present the risks associated with long-term cell culture (bacterial or fungal contaminations, muta- or tumorigenesis). Combined with a permissive regulatory framework in some countries, the use of SVF has regrettably been diverted by private clinics with little interest in studying the efficacy and mechanisms of action of this therapy, which has slowed its development. This was particularly observed in the United States, which had an unusually low number of publications on the topic [95] whereas a very large number of clinics offer these unproven therapies [96, 97]. However, this situation is about to change, with the end of the Food and Drug Administration discretion policy period which should enforce manufacturers, clinics, and health care practitioners compliance with the new guidance on the regulatory framework for regenerative medicine therapies and ensure a greater safety for the use of these therapies [98].

The use of SVF has been reported in many indications and it may represent a promising adjunctive therapy for patients with diseases for which current and conventional therapies are inadequate. The variety of these indications was recently reviewed by Andia et al. who analysed 71 published clinical studies evaluating SVF [99]. However, more than 65% of them were case series with a low level of evidence, and only 16% of them were randomised clinical trials. To confirm this analysis, we searched for clinical trials on clinicaltrial.gov database in May 2023 (search strategy with the key words: Stromal vascular fraction OR SVF OR ADSVF OR ADRC OR ADSC), which allowed us to identify 169 clinical trials evaluating SVF injection (Fig. 2B). The most common indications, according to these studies, were orthopaedic disorders (50.7% and 28.9% for published studies and clinical trials, respectively), then chronic wounds (14% and 24.1%) and cardiovascular and pulmonary diseases (12.6% and 13.9%) (Fig. 2B). Other frequent indications were urogenital (5.6% and 9.6%), neurological (5.6% and 4.8%), and autoimmune diseases (5.6% and 11%), which often overlapped with chronic wound treatment. These data are consistent with those from other studies [14, 99] and demonstrate the increasing interest of SVF for the treatment of inflammatory, degenerative or non-regenerative, autoimmune or cardiovascular diseases. Local sites of injection were more frequent than intravascular, with a predominance of sub-cutaneous, intra-articular, intra-fistula and intramuscular injection (Fig. 2D). Nevertheless, SVF is still a new therapy under development and clinical trials were mostly pilot studies in phase I (73% versus 32.9% for all clinical trials), and phase II and III appeared only recently (23% versus 42.4% and 4% versus 24.7%, respectively) (Fig. 2E). Furthermore, several SVF clinical trials were not completed (39% versus 15%), closed prematurely or withdrawn (23.3% versus 4.3%) (Fig. 2F). None of these closures were due to a safety issue.

Some recent studies reviewed adverse events in patients receiving “unproven or unapproved” stem cell therapies reported in scientific publications, clinical case reports and also mass media publications, and showed an increased number of severe complications and hospitalisations compared to conventional therapies [100, 101]. Conversely, very few treatment‐related adverse events were noted during clinical trials, demonstrating the safety of this procedure. Recently, clinical study results published for both SVF and ADSC were reviewed and showed that the most frequent and severe adverse events were immunological and thromboembolic. They concerned predominantly ADSC, which were more frequently used in an allogeneic context and via IV injection than SVF [95]. Indeed, the in vitro expansion of ADSC led to an increase in cell size, which significantly increased the risk of vascular obstruction and cerebral or myocardial stroke [102]. The injection of SVF directly at the lesion site or within organs seems to be a safer way to use this therapy.

Intramuscular injections of SVF were clinically used as a local route for the treatment of muscle sequelae [103, 104], allowing an improvement in muscle strength, or for the treatment of limb ischaemia [105, 106], highlighting its proangiogenic effects. In these studies, the safety of this route was confirmed. Furthermore, IM injection led to the release of paracrine effectors in blood circulation and could be an alternative to other injection sites (intrathecal, intra-articular) [107]. Another advantage of this route was the increased dwell time of the injected cells, increasing from days to months the persistence in the body of these cells [108]. Even if they remained in the muscle, they still responded to distant inflammatory signals and acted on distant sites [109]. IM injection of SVF could lead to prolonged clinical efficacy compared to other routes, in both injected and non-injected muscles.

### Potential mechanisms of action of stromal vascular fraction in myositis

**Fig. 3:**
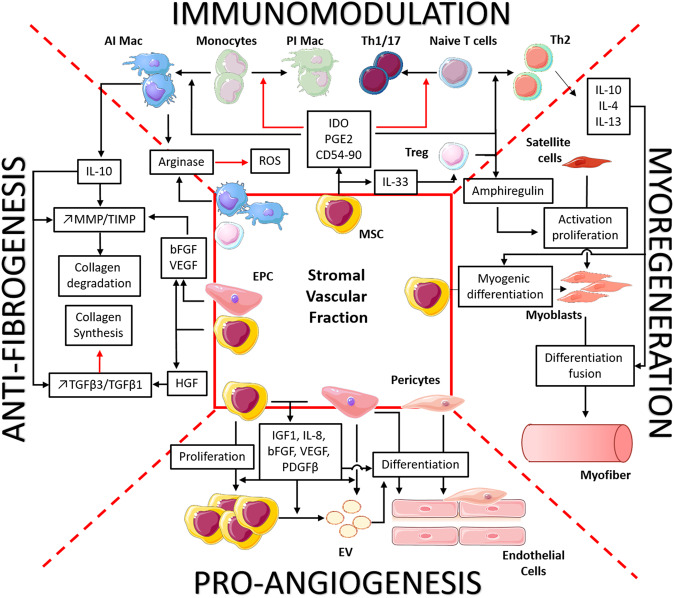
Stromal vascular fraction induces immunomodulatory, myoregenerative, anti-fibrotic and proangiogenic effects by different cell types. Immunomodulatory capacity is mostly mediated by MSC, through reduction of Th1/Th17 cells, by inhibiting their differentiation from naive T cells and increasing differentiation to Th2 cells via IDO, PGE2, CD54-CD2 interaction. MSC also differentiate monocytes into anti-inflammatory macrophages (AI Mac) by trapping them into an anti-inflammatory environment through CD90-CD11b interaction. Adipose tissue Treg and Macs add themselves to immune cells present in the muscle to reduce inflammation. These cells also release IL-10, which changes the MMP/TIMP balance in favour of collagen degradation. This ratio is also changed by bFGF and VEGF, release by EPC and MSC. MSC also release HGF which changes, like IL-10, the TGFβ3/1 balance to reduce collagen synthesis. Along with EPC, they also release IGF1, bFGF, VEGF, PDGFβ and extracellular vesicles (EV), which act at different levels to differentiate EPC and pericytes into endothelial cells and thus reinforce the vascular network. Finally, MSC could differentiate themselves into myoblasts and replenish the muscle cell supply, exhausted by chronic inflammation. Amphiregulin and anti-inflammatory cytokines IL-10, IL-4 and IL-13 released by immune cells participate in the proliferation, differentiation and fusion of these cells.

As seen above, intramuscular injection of SVF is known to be safe and has promising clinical effects in both autoimmune and muscular diseases. Its potential interest in myositis treatment is based on four properties, but mechanisms of action are not fully known (Fig. 3).

First, the immunomodulatory effect of SVF is supported mainly by three cell populations: AD-MSC, macrophages and Treg cells. AD-MSC immunomodulatory capacities are similar or higher to those of BM-MSC according to different studies [110, 111] and have already been tested in vitro [112] and in many in vivo models [113]. Even if the mechanisms involved are not fully understood for these cells, several studies support their effects on T-cell activation, proliferation and differentiation from Th1 cells into Th2 cells, through soluble factors like PGE2 and IDO [114–116] and direct interactions via CD54/CD2 and CD58/CD11a, which increase IL-10 production [117]. They also induce Treg proliferation, via TGFβ and IL-33 secretion [118, 119], and few adipose tissue Tregs are present in SVF. MSC also act on macrophage anti-inflammatory switch, in part by the secretion of PGE2 and IDO [120] and the interaction of CD90 and CD11b trapping monocytes and macrophages into an anti-inflammatory niche [121]. Recent studies have shown that the interactions between MSC and pro-inflammatory cells enhance the immunosuppressive capacities of MSC. Indeed, these authors observed that IFNγ and TNFα secreted by Th1 lymphocytes or CD54 expressed by pro-inflammatory macrophages increased IDO activities [122, 123]. SVF also contains macrophages which exhibit anti-inflammatory activities through the secretion of high levels of IL-10 and IL-1 decoy receptors [124] that attenuate TNFα inflammatory signals via activation of STAT3 [125, 126], and modulation of inflammatory gene transcription rates [127]. The modification of the balance between Arg-1 and iNOS activities, which both use L-arginine as a substrate, leads to decreased ROS production, and thus to reduced oxidative stress and destruction of myofibres.

ADSC and SVF can also act on fibrosis via their immunomodulatory effects. Indeed, by reprogramming immune cells into anti-inflammatory cells, they increase the expression of IL-10, which presents several anti-fibrotic properties: inhibition of neutrophil and macrophage invasion and ROS release [128], down-regulation of TGFβ1 expression [129], up-regulation of MMP and down-regulation of collagen expression [130]. Preclinical and clinical studies suggest that SVF anti-fibrotic effects are strongly related to the secretion of HGF by MSC during inflammatory responses, as evidenced by clinical and histological parameters [131, 132]. Indeed, through the paracrine effect of this factor, SVF and ADSC reduce the expression of TGFβ1 and thus the differentiation of collagen type I/III producing cells (fibroblasts) and alpha-Smooth Muscle Actin producing cells (myofibroblasts). ADSC also induce a significant increase in TGFβ3, which reduces the expression of these genes, and stimulates MMP-1, -2 and -3 expressions, which increase fibrotic molecule degradation. The change in the TGFβ1/TGFβ3 and MMP-2/TIMP-2 ratio tips the scales in favour of an anti-fibrotic effect [133, 134]. MMP expression is also stimulated by proangiogenic factors, like bFGF or VEGF, to degrade extracellular matrix and prepare neo-angiogenesis [135].

Indeed, SVF is known to express high levels of IGF1, IL-8, Platelet-Derived Growth Factor-beta (PDGFβ), bFGF and VEGF, and to have robust angiogenic and vasculogenic activities demonstrated both in vitro and in vivo in a hind limb ischaemia model [136]. These growth factors help to maintain a vascular-like micro-environment that supports MSC differentiation into endothelial cells, and thus participate in angiogenesis and vascular repair during muscular regeneration [137]. Traktuev et al. demonstrated that VEGF helps the migration of MSC and promotes the secretion of PDGFβ by EPC, which enable MSC to proliferate [138, 139]. PDGFβ is well-known for its action during vascular development [140] but also plays a role in the proangiogenic properties of SVF, by inducing the secretion of proangiogenic extracellular vesicles by both MSC and EPC [141, 142]. These extracellular vesicles contain proangiogenic molecules such as c-KIT and Stem cell factor, which participate in the recruitment of EPC and their differentiation into endothelial cells [143]. PDGFβ secretion by EPC also induces pericyte recruitment [144] which is known to play an essential role in angiogenesis regulation [145].

SVF, through its immunomodulatory properties, acts on both chronic inflammation and muscle repair via cytokine release (mainly IL-4 and IL-13). Moreover, growth factors secreted by stromal cells may have a positive effect on muscle regeneration, and some of them are currently under evaluation in the management of muscle disorders, such as sarcopenia [146]. But one advantage to using cell therapy, rather than hormones or cytokines, could be its ability to differentiate in situ depending on its cellular environment. This could strengthen and help satellite cells to replace defective cells. The conversion of ADSC or SVF to a myogenic phenotype has been obtained in vitro by addition of inductive media, containing horse serum and hydrocortisone. This leads to the expression of the myogenic transcription factors Myod1 and myogenin and then the fusion and formation of multinucleated cells expressing the myosin heavy chain [147–149]. Based on histological evidence, ADSC fuse to form multinucleated myotubes in vitro. In their study, Di Rocco et al. showed that ADSC and SVF cells were able to differentiate into skeletal muscle cells when cultured in the presence of differentiating primary myoblasts [150]. Furthermore, the conversion of SVF to a myogenic phenotype is enhanced by myogenic environment in the absence of cell-cell contacts (transwell culture) and even in absence of muscle cells but to a lesser extent. This myogenic conversion has also been demonstrated in vivo by several studies. In a lagomorphic model of muscular injury induced by cardiotoxin, the intramuscular injection of short-term cultured (3 days) SVF cells induced an increase in muscle mass and functional capacities [151]. The myogenic differentiation of SVF and fusion with muscular cells have been demonstrated using SVF genetically modified to express β-galactosidase or GFP, showing evidence of the contribution of SVF cells to muscular regeneration in vivo with 20% of GFP-positive fibres in the total area of sections from treated hind limbs [150, 151]. This contribution could be enhanced by pretreating SVF with anti-inflammatory cytokine IL-4 or SDF1 before use to increase the myogenic capacity of ADSC in vitro and in vivo [152]. To finish, it has also been reported that injection of human ADSC into immunocompetent mdx mice resulted in a substantial expression of human dystrophin in both injected and adjacent muscle, revealing the spread of cells to other muscles [153].

## Conclusion

MSC-based therapies have shown interesting effects in the treatment of refractory myositis, but their clinical use remains limited, especially for those extracted from adipose tissue. However, SVF, easily harvested from this tissue, could be beneficial for patients thanks to its properties combining an immunomodulatory effect and a response to the main muscular complications of myositis. While published cases report only IV infusion of MSC in myositis treatment, IM injection of SVF seems to be an interesting alternative, providing both local and systemic effects. Taken together, the evidence reviewed here seems to predict a potential benefit of SVF in myositis treatment. However, these findings also highlight the need for preclinical studies and clinical trials to better understand the mechanisms of this therapy and to optimise the practical modalities to ensure its safety and efficacy.
